# Predicting the Outcome of Sjogren’s Syndrome-Associated Non-Hodgkin’s Lymphoma Patients

**DOI:** 10.1371/journal.pone.0116189

**Published:** 2015-02-27

**Authors:** Aristea Papageorgiou, Dimitrios C. Ziogas, Clio P. Mavragani, Elias Zintzaras, Athanasios G. Tzioufas, Haralampos M. Moutsopoulos, Michael Voulgarelis

**Affiliations:** 1 Department of Pathophysiology, School of Medicine, University of Athens, Athens, Greece; 2 Department of Physiology, School of Medicine, University of Athens, Athens, Greece; 3 Department of Biomathematics, School of Medicine, University of Thessaly, Larissa, Greece; University of Bari Medical School, ITALY

## Abstract

**Background:**

Non-Hodgkin's lymphoma (NHL) development in Sjögren’s syndrome (SS) remains a potentially lethal complication and efforts should focus on the identification of predictors that could aid in appropriate therapeutic decisions.

**Methods:**

In order to identify potential prognostic factors for outcome in SS-associated NHL, we retrospectively analyzed a cohort of 77 patients, diagnosed with NHL according to WHO classification criteria and meeting the American-European Consensus Classification (AECC) criteria for SS and examined the effect of SS-activity (defined as the EULAR SS disease activity index-ESSDAI) in the prognosis of SS-related NHLs, as defined in terms of overall and event-free survivals (OS and EFS). An event was defined as lymphoma relapse, treatment failure, disease progression, histological transformation or death. The effect of NHL clinical and laboratory characteristics was also investigated.

**Results:**

MALT lymphomas constituted the majority (66.2%) of lymphomas. During the follow-up (median = 57.93 months), the 5-year OS was 90.91% (95% CI: 82.14–95.80%) and the EFS was 77.92% (95% CI: 67.37–85.82%). Patients with high ESSDAI score at lymphoma diagnosis had a greater risk for death (OR = 5.241, 95% CI: 1.034–26.568) or for event (OR = 4.317, 95% CI: 1.146–9.699, p = 0.008). These patients had also significantly worse EFS (HR = 4.541, 95% CI: 1.772–11.637) and OS (HR = 5.946, 95% CI: 1.259–28.077). In addition, post-chemotherapy ESSDAI improvement was significantly lower in patients who had experienced an event (p = 0.005). An unfavorable International prognostic index (IPI) score (high-intermediate/high) was associated with high risk of death and event (OR = 13.867, 95% CI: 2.656–72.387 and OR = 12.589, 95% CI: 3.911–40.526, respectively), worse EFS (log-rank p<0.001, HR = 8.718, 95% CI: 3.477–21.858), as well as with worse OS (log-rank p<0.001, HR = 11.414, 95% CI: 2.414–53.974). After adjustment for identified risk factors, IPI score retained a significant prognostic role following by a strong effect of ESSDAI in survival outcomes.

**Conclusions:**

At the point of NHL diagnosis, IPI and ESSDAI might be proved useful predictive tools in SS-associated lymphoma prognosis, directing to a more patient-tailored approach.

## Introduction

Primary Sjögren’s syndrome (SS) has been generally acknowledged as a chronic benign autoimmune disease characterized by slowly progressive signs and symptoms of a destructive, mainly salivary exocrinopathy. However, in more than 20–40% of patients the disease extends beyond the exocrine glands, manifested either by epithelial lymphocytic invasion of the lung, liver or kidney or by immune complex-mediated phenomena such as skin vasculitis, peripheral neuropathy, glomerulonephritis and low serum C4 levels, all of which have previously been shown to confer increased risk for non-Hodgkin’s lymphoma (NHL) development [1–3].

In the setting of SS, NHL is the most detrimental complication, affecting 5–10% of these patients [1,4–7]. Indolent extranodal marginal zone B-cell lymphoma of the mucosa-associated lymphoid tissue (MALT) has previously been identified as the most common SS-associated lymphoma subtype, whilst MALT lymphoma (MALTL) in the salivary gland is consistently associated with underlying SS [3,8–20]. The corresponding age and sex-adjusted standardized mortality ratio of SS with and without NHL is 3.25 and 1.08 respectively, suggesting a shorter life expectancy in the former group [21]. Consequently, lymphoma development in SS remains a potentially lethal complication, and efforts should focus on the identification of predictors that could aid in appropriate therapeutic decisions. The long-standing question is whether certain clinical, and laboratory characteristics at lymphoma diagnosis can adequately predict the outcome and promptly identify those SS-associated lymphoma patients at high risk of death. The main objective of the present study was to identify potential prognostic SS- or lymphoma-related factors in a cohort of SS-related lymphoma patients.

## Materials and Methods

### Study cohort

We evaluated the medical records of all consecutive patients with an initial diagnosis of primary SS in the Department of Pathophysiology (School of Medicine, University of Athens) from 1993 to September 2013. Seventy-seven patients, diagnosed with NHL according to WHO classification criteria and meeting the American-European Consensus Classification (AECC) criteria for SS, were considered eligible for this study [22,23]. Fifteen of these patients were referred to the hematology unit of our department after NHL diagnosis, from other Greek Rheumatology centers, for further management. None of the SS-associated NHL lymphomas were developed before SS diagnosis, nor were they associated with hepatitis C virus infection. In parallel, we pooled 167 consecutive SS patients without lymphoma from a cohort of 712 SS patients that were followed in our department and used them as controls.

### Data Collection and Definition of Outcomes

The following parameters at lymphoma diagnosis for all SS-associated NHL and SS-non lymphoma cases (where applicable) included: age, sex, NHL subtype, Eastern Cooperative Oncology Group (ECOG) performance status (PS) [24], Ann Arbor stage (I-IV) [25], number and type of involved sites, International Prognostic Index (IPI) [26], splenomegaly, lymphadenopathy, presence of B symptoms, Helicobacter Pylori status, hemoglobin levels, white blood cells and differential cell count, platelet count, serum lactate dehydrogenases (LDH), β2-microglobulin levels, serum protein electrophoresis, serum immunoglobulin quantification, rheumatoid factor (RF), and serum complement C3 and C4 levels. Anemia was defined as a hemoglobin level <12 g/dL; hypocomplementemia as C4<16mg/dL and C3 <75 mg/dL; neutropenia and lymphopenia as neutrophil levels <1500/mm^3^ and lymphocyte levels <1000/mm^3^, respectively. Patients were distributed among the IPI risk groups according to the published data [26].

All included lymphoma and control cases in our study had a salivary gland biopsy at SS diagnosis. A blinded pathologist quantified local inflammatory infiltration, based on the number of foci present in the glands, classified as the focus score (FS) [27]. In the lymphoma group a salivary gland biopsy was performed in all patients at lymphoma diagnosis. The patients with salivary gland lymphomatous involvement were reevaluated with a second salivary gland biopsy 6 months after treatment completion.

In order to comprehensively describe the systemic manifestations and quantify the activity of primary SS, we used the European League Against Rheumatism (EULAR) SS disease activity index (ESSDAI), the total score of which was estimated at lymphoma diagnosis as well as at SS diagnosis for SS-associated NHL cases and at SS diagnosis for SS-non lymphoma controls [28]. We also calculated the change/improvement in the total ESSDAI six months after first-line lymphoma treatment. During follow-up, we assessed SS-associated NHL patients for treatment response at 4, 6, and 12 months after initiation of treatment and thereafter every 6 months, and obtained data on response, treatment failure, histologic transformation to a high-grade lymphoma, relapse, cause of death, as well as lymphoma and SS status at the last follow-up. Complete remission (CR), relapse and progression of lymphoma were defined according to previously published reports [29]. An event was defined as lymphoma relapse, treatment failure, disease progression, histological transformation or death after NHL diagnosis. According to the National Cancer Institute (NCI) criteria, overall survival (OS) was calculated from the time of diagnosis of lymphoma to the time of death from any cause or last follow-up and event-free survival (EFS) was estimated from the time of diagnosis of lymphoma to the time of the first event or the last follow-up. All patients provided written, informed consent and the study was approved by the Ethics Committee of Laiko General Hospital.

### Statistical analysis

All analyses were done with use of data obtained until 30 September 2013. For categorical variables, data are presented as frequencies with their corresponding 95% confidence intervals (CI), and for continuous variables as means with standard deviation (SD) or as median with observed range (minimum-maximum). The 95% CIs of proportions were computed by modified Wald method. To compare categorical variables, we used the x^2^ test or the Fischer’s exact test where appropriate. To compare continuous variables, the Mann-Whitney (two-tailed) test was applied. Paired comparisons of over-time changes in continuous and categorical variables were performed with the Wilcoxon’s matched pairs test and the McNemar’s test, respectively. Survival curves were plotted and time-to-event analyses were estimated by the method of Kaplan-Meier; differences between curves were analyzed using the log-rank test. The median follow-up times were computed by the reverse Kaplan-Meier method. Unadjusted and adjusted hazard ratios (HR) with the respective 95% CIs were estimated using univariate and multivariate Cox regression analysis, respectively. The multivariate Cox regression analysis examined the effect on OS and EFS after adjustment for identified baseline (at lymphoma diagnosis) prognostic parameters. Also, unadjusted and adjusted odds ratios (OR) with the respective 95% CIs for examining the effects of OS and EFS without considering the time effect were estimated using logistic regression. Adjustment was performed for identified baseline (at lymphoma diagnosis) prognostic parameters.

To include total ESSDAI and IPI scores in our analysis, we grouped SS-associated NHL patients, using the median value of total ESSDAI score of our cohort as the dichotomous threshold: high SS disease activity group (High ESSDAI score>median value) and low SS disease activity group (low ESSDAI score<median value); and modifying the IPI risk subgroups as following: low/low-intermediate IPI risk group (= 0–2 points) and high-intermediate/high IPI risk group (= 3–4 points).

To address the realistic issue of missing data potentially emerging from the retrospective pattern of our study, frequent communication, wherever possible, was made with the patients themselves or their relatives by phone, email or fax thereby enabling us to complete our questionnaires and gather laboratory results. For all data-points collected, completeness of data exceeded 95%.

Statistical analyses were conducted using SPSS software package version 21 (Computing Resource Centre, Santa Monica, CA, USA) and GraphPad Prism software (GraphPad Software, Inc. La Jolla, CA, USA). Statistical significance was defined as a p-value of less than 0.05 for all comparisons.

## Results

### Study population

Baseline characteristics of 77 SS-patients (5 males and 72 females) with confirmed histologic diagnosis of NHL, their first-line treatments and CR rates, according to lymphoma subtypes are presented in Table 1. The median age at diagnosis of lymphoma was 58 years (range 28–90 years), while the median time from SS diagnosis to lymphoma diagnosis was 65.8 months (range, 0–456.2 months). The median follow-up from SS-associated lymphoma diagnosis up to the last visit/update was 57.93 months (4.83 years) with 26% of cases were followed up for more than seven years. Among 77 patients, 32 (41.5%) had received immunosuppressive treatment before lymphoma diagnosis. More specifically, immunosuppression was low dose prednisolone alone (13 cases), D-penicillamine (1 case), a combination of methotrexate and prednisolone (8 cases), a combination of cyclophosphamide and prednisolone (3 cases), a combination of azathioprine and prednisolone (2 cases), and monotherapy with rituximab (5 cases). There was no difference on the type of lymphoma or its evolution between patients who received immunosuppressive treatment before lymphoma diagnosis compared to those who did not.

**Table 1:** Characteristics of SS-associated lymphoma patients, first-line treatments and CR rates, according to NHL subtypes.

	Total SS-associated lymphoma patients	MALTL	NMZL	DLBCL	Other lymphomas
**Demographics**					
Number (no) of patients	77	51	8	12	6
Female sex, no	72	47	7	12	6
Age, years					
Median (range)	58 (28–90)	55 (28–76)	56 (36–90)	69 (44–81)	62 (52–80)
Mean (SD)	56.16 (13.57)	53.12 (12.55)	56.25 (16.82)	64.83 (13.12)	64.50 (9.50)
Median time from SS to lymphoma diagnosis, months (range)	65.80 (0–456.2)	61.53 (4.47–446.0)	91.57 (0–251.3)	97.54 (13.53–456.2)	13.18 (2.2–72.97)
Median follow-up, months^§^	57.93	59.20	30.17	47.52	90.42
ECOG PS≤1, no (%)	72 (93.5)	51 (100)	4 (50)	12 (100)	5 (83.33)
**Presentation of SS systematic manifestations in ESSDAI domains**					
Skin, no (%)	40 (51.9)	23 (45.1)	6 (75.0)	9 (75.0)	2 (33.3)
Pulmonary, no (%)	17 (22.1)	12 (23.5)	1 (12.5)	4 (33.3)	0 (0.0)
Renal, no (%)	19 (24.6)	9(17.6)	6 (75.0)	3 (25.0)	1 (16.7)
Articular, no (%)	74 (96.1)	50 (98.0)	7 (87.5)	11 (91.6)	6 (100)
Muscular, no (%)	0 (0.0)	0 (0.0)	0 (0.0)	0 (0.0)	0 (0.0)
Peripheral neuropathy, no (%)	20 (26.0)	9 (17.6)	4 (50)	6 (50)	1 (16.7)
CNS involvement, no (%)	1 (1.3)	1 (2.0)	0 (0.0)	0 (0.0)	0 (0.0)
Salivary/parotid enlargement, no (%)	52 (67.5)	38 (74.5)	4 (50)	6 (50)	4 (66.7)
Constitututional, no (%)	30 (39.0)	14 (27.5)	7 (87.5)	7 (58.3)	2 (33.3)
Hematological, no (%)	58 (75.3)	35 (68.6)	6 (75.0)	12 (100)	5 (83.3)
Lymphadenopathy, no (%)	47 (61.0)	27 (52.9)	8 (100)	8 (66.7)	5 (83.3)
Biological, no (%)	70 (90.9)	46 (90.2)	8 (100)	12 (100)	4 (66.7)
**SS activity/Total ESSDAI score**					
Mean (SD)	10.31 (4.02)	9.49 (3.83)	14.00 (3.12)	12.17 (4.02)	8.67 (3.20)
Median (range)	10 (4–20)	9.00 (4–20)	14.00 (9–19)	12.50 (6–17)	7.50 (6–13)
**Other clinical features**					
B-symptoms, no (%)	5 (6.5)	0 (0)	1 (12.5)	2 (16.7)	2 (33.3)
Splenomegaly, no (%)	12 (15.6)	6 (11.8)	6 (75)	1 (8.3)	3 (50)
Palpable purpura, no (%)	33 (42.9)	22 (23.6)	4 (50)	7 (58.3)	0 (0)
**Ann Arbor staging, no (%)**					
Stage I	31 (40.3)	29 (56.9)	0 (0)	2 (16.6)	0 (0)
Stage II	9 (11.7)	3 (5.9)	0 (0)	4 (33.3)	2 (33.3)
Stage III	6 (7.8)	0 (0)	4 (50)	2 (16.6)	0 (0)
Stage IV	31 (40.3)	19 (37.3)	4 (50)	4 (33.3)	4 (66.7)
**IPI score**, no (%)					
Low risk (0–1 points)	30 (38.9)	28 (54.9)	0 (0)	2 (16.6)	0 (0)
Low-intermediate risk (2 points)	24 (31.2)	14 (27.5)	4 (50)	3 (25)	3 (50)
High-intermediate risk (3 points)	18 (23.4)	5 (9.8)	3 (37.5)	7 (58.3)	3 (50)
High risk (4–5 points)	5 (6.5)	4 (7.8)	1 (12.5)	0 (0)	0 (0)
**Hematological sites**					
BM involvement, no (%)	21 (27.3)	15 (29.4)	0 (0)	2 (16.7)	4 (66.7)
Extranodal sites **≥1,** no (%)	29 (37.7)	25 (49)	0 (0)	2 (16.7)	2 (33.3)
**Laboratory findings**					
Anemia (Hgb<12mg/dl), no (%)	34 (44.2)	20 (39.2)	4 (50)	7 (58.3)	3 (50)
Leucopenia (WBC<4000/mm^3^), no (%)	18 (23.4)	13 (25.5)	3 (37.5)	1 (8.3)	1 (16.7)
Lymphopenia (LYM<1000/mm^3^), no (%)	32 (41.6)	21 (41.2)	4 (50)	5 (41.7)	2 (33.4)
Elevated LDH (>220mg/dl), no (%)	42 (54.5)	25 (49.0)	6 (75)	7 (58.3)	4 (66.7)
Elevated β_2_-microglobulin, no (%)	38 (49.4)	24 (47.1)	5 (62.5)	5 (41.7)	4 (66.7)
Low C3 levels (<75 mg/dL), no (%)	10 (13.0)	8 (15.6)	2 (25)	0 (0)	0 (0)
Low C4 levels (<16 mg/dL), no (%)	36 (46.8)	24 (47.1)	5 (62.5)	6 (50)	1 (16.7)
PositiveANA, no (%)	64 (83.1)	46 (90.2)	8 (100)	8 (66.7)	2 (33.4)
Positive Ro, no (%)	61 (79.2)	43 (84.3)	7 (87.5)	9 (75)	2 (33.4)
Positive La, no (%)	43 (55.8)	31 (60.8)	4 (50)	7 (58.3)	1 (16.7)
Positive RF, no (%)	51 (66.2)	36 (70.6)	7 (87.5)	7 (58.3)	1 (16.7)
Hypergammaglobulinemia, no (%)	35 (45.5)	26 (51)	4 (50)	3 (25)	2 (33.4)
Hypogammaglobulinemia, no (%)	7 (9.1)	2 (3.9)	2 (25)	1 (8.3)	2 (33.4)
Monoclonal band, no (%)	24 (31.2)	16 (31.4)	5 (62.5)	1 (8.3)	2 (33.4)
Cryoglobulinemia, no (%)	26 (33.8)	18 (35.3)	5 (62.5)	3 (25)	0 (0)
**First-line treatment and CR rates**					
Anti-CD20 monotherapy (Rituximab), no	14	14			
CR, no (%)	10 (71.4)	10 (71.4)			
Antibiotics, no	4	4			
CR, no (%)	2 (50)	2 (50)			
Single agent, no					
-alkylating agent, CHL or C	4	CHL: 1	C: 1		CHL: 2
CR, no (%)	0 (0)	0 (0)	0 (0)		0 (0)
-purine analog, 2Cda	3	1	1		1
CR, no (%)	3 (100)	1 (100)	1 (100)		1 (100)
Polychemotherapy, no	9	COP: 2	COP: 2 CHOP: 1	CHOP: 1	CHOP: 1 COP: 1 CHOP/ESHAP: 1
CR, no (%)	4 (44.4)	1 (50)	COP: 1 (50) CHOP: 0	1 (100)	CHOP: 0 (0) COP: 1 (100) CHOP/ESHAP: 0 (0)
Rituximab plus Chemotherapy, no	32	RCHOP: 6 RF: 10 RCOP: 1 RC: 2	RC: 1 RCHOP: 1	RCHOP: 11	
CR, no (%)	22 (68.8)	RCHOP: 5 (83.3) RF: 7 (70) RCOP: 1 (100) RC: 1 (50)	RC: 0 (0) RCHOP: 1(100)	RCHOP: 7 (63.6)	
Irradiation, no (%)	1	1			
“Wait and see” policy, no (%)	10	9	1, denied		
Total CR after first-line treatment, no (%)	41 (61.2)	28 (68.3)	3(37.5)	8 (66.7)	2 (33.3)

**Abbreviations**: SS = Sjögren's syndrome, MALTL = Mucosa-associated lymphoid tissue lymphoma, DLBCL = diffuse large B-cell lymphoma, NMZL = nodal marginal zone lymphoma, ESSDAI = EULAR Sjögren's syndrome disease activity index, IPI = International Prognostic Index, BM = Bone Marrow, SD = standard deviation, 2Cda = 2-chlorodeoxyadenosine, CHL = chlorambucil, CHOP = cyclophosphamide, doxorubicin, vincristine, prednisone, COP = cyclophosphamide, vincristine, prednisone, R = anti-CD20 (rituximab), C = cyclophosphamide, R-CHOP = rituximab, cyclophosphamide, doxorubicin, vincristine, prednisone, RF = rituximab, fludarabine, ESHAP = etoposide, methylprednisolone, cytarabine, cisplatin, CR = complete remission.

§Median follow-up times were computed by the reverse Kaplan-Meier method.

MALTLs constituted the majority (51/77, 66.2%) of NHL subtypes, followed by diffuse large B-cell lymphomas (DLBCL) (12/77, 15.6%) and nodal marginal zone lymphomas (NMZL) (8/77, 10.4%). Compared to patients with DLBCL, MALTL patients were significantly younger (median age was 55 versus 69 years respectively, p = 0.007), and developed lymphoma much earlier (median time from SS diagnosis to MALTL development was 65.80 versus 97.54 months respectively, p = 0.004).

At the time of NHL diagnosis, ECOG performance status of patients was fairly good (93% of patients had a PS≤1), 52% of patients had a limited disease (I and II), according to Ann Arbor staging, and 70.2% of patients belonged to the low-risk group and low-intermediate risk IPI groups (Table 1). In the entire cohort, 21 patients (27.3%) had bone marrow (BM) involvement and 29 patients (37.7%) presented with several extra-nodal involvement at diagnosis.

### Systemic manifestations and activity of Sjögren's syndrome

Total ESSDAI score was used to quantify SS-activity. For SS-associated NHL patients mean total ESSDAI score (SD) was 10.31 (4.02) at lymphoma diagnosis, while at SS diagnosis was 3.12 (1.74). In comparison with the control group of 167 SS patients without lymphoma (8 males and 159 females, median age, range: 54, 29–79 years, mean total ESSDAI±SD: 2.86±1.74), the total ESSDAI score of our SS-associated lymphoma cohort at time of SS diagnosis, was similar to the ESSDAI score of SS patients that did not develop latterly NHL (p = 0.279). In the NHL subgroup analysis, MALTL patients had significantly lower total ESSDAI score (mean±SD, 9.49±3.83) compared with DLBCL patients (12.17±4.02) (p = 0.037) and NMZL patients (14±3.12) (p = 0.003) (Table 1).

Skin vasculitis (51.9%, 40/77), articular involvement (98.7%, 76/77), parotid gland enlargement (70.1%, 54/77), hematological manifestations (75.3%, 58/77), lymphadenopathy (71.4%, 55/77) and biological parameters (90.9%, 70/77) were common (>50%) in SS-associated lymphoma patients compared to SS-non lymphoma matched controls. Furthermore, twenty NHL patients (26%) developed peripheral neuropathy and 33 NHL patients (42.9%) displayed palpable purpura. Among the hematological and biological findings, anemia, lymphopenia, the presence of monoclonal immunoglobulin and cryoglobulinemia were detected more often in NHL patients (Table 1). No statistically significant differences in the aforementioned clinical manifestations were detected between NHL subgroups.

Six-months after NHL treatment, total ESSDAI score and biological parameters (such as cryoglobulinemia, hypergammaglobulinemia, RF, C3 and C4 levels) were re-assessed (Table 2). Gamma globulin levels, C4 levels, RF titers and the presence of cryoglobulinemia were significantly improved (p<0.001, p = 0.004, p<0.001, p<0.001 respectively). In addition, the majority of the presented ESSDAI items and the total ESSDAI score showed significant improvement (Table 2), but disease activity in treated NHL patients was still higher compared to non-lymphoma SS cases (p = 0.001). Among 42 salivary MALT lymphoma cases, complete remission was achieved after treatment in 22 (52.38%, 95%CI: 37.72–66.64%). Focus score, evaluated 6 months after treatment completion, was also significantly improved compared to FS at lymphoma diagnosis in these patients (mean±SD, 3.60±1.93 vs mean±SD, 5.73±2.21 respectively) (p = 0.002). On the other hand NHL patients without salivary gland involvement exhibited a FS of 3.39 (3.93) at lymphoma diagnosis. Of interest FS of salivary gland biopsies in all lymphoma patients at SS diagnosis (mean±SD, 3.28±2.57) was significantly higher compared to the FS of control group at SS diagnosis (mean±SD, 1.58±1.57) (p<0.001).

**Table 2:** Differentiation of laboratory findings, SS systemic manifestations and SS activity, according to total ESSDAI score, 6-months after NHL treatment.

	At lymphoma diagnosis	After lymphoma treatment	P-value#
**Laboratory findings**			
Positive RF, no (%)	51 (66.2)	18 (23.4)	<0.001
Low C3 levels (<75 mg/dL), no (%)	10 (13.0)	8 (10.4)	0.774
Low C4 levels (<16 mg/dL), no (%)	36 (46.8)	20 (25.9)	0.004
Hypergammaglobulinemia, no (%)	35 (45.5)	7 (9.1)	<0.001
Cryoglobulinemia, no (%)	26 (33.8)	8 (10.4)	<0.001
**Presentation of SS systemic manifestations in ESSDAI domains**			
Skin	40 (51.9)	28 (36.4)	<0.001
Pulmonary	17 (22.1)	14 (18.2)	0.250
Renal	19 (24.6)	17 (22.1)	0.500
Articular	74 (96.1)	42 (54.5)	<0.001
Muscular	0 (0.0)	0 (0.0)	1.000
Peripheral neuropathy	20 (26.0)	17 (22.1)	0.375
CNS	1 (1.3)	1 (1.3)	1.000
Glandular	52 (67.5)	44 (57.1)	0.008
Constitutional	30 (39.0)	23 (29.9)	0.016
Haematological	58 (75.3)	11 (14.3)	<0.001
Lymphadenopathy	47 (61.0)	17 (22.1)	<0.001
Biological	70 (90.9)	18 (23.4)	<0.001
**Activity of SS systemic manifestations**			
Total ESSDAI score, mean (SD)	10.31 (4.02)	4.07 (3.02)	< 0.001

**Abbreviations**: ESSDAI = EULAR Sjögren's syndrome disease activity index, RF = rheumatoid factor.

#For comparisons of proportions, P-value was calculated with McNemar’s test and for comparison of total ESSDAI scores (continuous variable)

P-value was calculated with Wilcoxon matched pairs test

### Outcomes and survival

During follow-up, 10 patients died, five suffered a relapse, two experienced progression/transformation, and five patients developed other hematological malignancies that included multiple myeloma (one), Hodgkin’s disease (one), T-cell NHL (two), and thymoma (one). Seven of the recorded deaths were attributed to treatment-related neutropenic sepsis; two deaths were the result of relapse after treatment failure, and one death was attributed to reasons that were not lymphoma or treatment-related. Table 3 summarizes the 22 SS-associated lymphoma cases that experienced an event during follow-up.

**Table 3:** Summary of 22 SS-associated NHL-patients that experienced an event during follow-up.

A/a	Age/Sex	Type	Stage	IPI	First line TX	EFS (months)	Event	Second line TX	Outcome/Comment
1	48/F	DLBCL	IV	Inter/High	R-CHOP	36.0	Relapse	R-CHOP	CR
2	75/F	DLBCL	III	Inter/High	R-CHOP	42.3	Death †		In CR, lymphoma related
3	61/F	MALTL	I	Low/Inter	R	35.3	Relapse	R-FMD	CR
4	54/F	NMZL	IV	Inter/High	COP	16.4	Relapse	2CDA	Death †, treatment related
5	76/F	MALTL	IV	High	CHL	0.3	Death †		Death †, treatment related
6	62/F	LPL	IV	Inter/High	CHL/B	85.3	Progression	CHOP	Death †, treatment related
7	57/F	MALTL	II	Low	RF	29.9	Death †		In CR, treatment related
8	58/F	NMZL	IV	Inter/High	C	37.8	Relapse	CHOP	Lost in follow-up
9	60/F	NMZL	IV	High	COP	80.1	Relapse	CHOP	Death †, treatment related
10	52/F	T-NHL	IV	Inter/High	CHOP/ ESHAP	43.8	Hodgkin’s disease	ABVD	CR
11	36/F	NMZL	III	Low/Inter	R-CHOP	61.7	Relapse	RF	CR
12	44/F	DLBCL	IV	Inter/High	CHOP	19	Relapse	R-ESHAP/ASCT	CR
13	81/F	DLBCL	II	Inter/High	R-CHOP	12.2	Relapse	R-CHOP	Death †, treatment related
14	57/F	MALTL	IV	Inter/High	R	22.8	Relapse	R-CHOP	CR
15	72/F	MALTL	IV	High	RF	70.9	Multiple myeloma	No therapy	CR
16	76/F	DLBCL	II	Inter/High	R-CHOP	9.3	Progression	R-ICE	Death †, treatment related
17	63/F	MALTL	I	Low/Inter	CHOP	41.7	Transformation	RF	CR
18	39/F	MALTL	IV	Low/Inter	R-CHOP	78.3	Thymoma	No therapy	CR
19	59/F	DLBCL	I	Low/Inter	R-CHOP	26.7	Relapse	R-ESHAP	CR
20	73/F	MALTL	IV	High	RF	10.2	Transformation	No therapy	T-LGL, Death †, lymphoma related
21	43/F	MALTL	IV	Low/Inter	R-CHOP	69.7	Relapse	R-2CDA/BDR	T-NHL, R-ESHAP/ASCT, CR
22	55/F	MALTL	IV	Low/Inter	R-CHOP	7.8	Death †		Death treatment-lymphoma-unrelated

**Abbreviations**: MALT = Mucosa associated lymphoid tissue, DLBCL = Diffuse large B-cell lymphoma, NMZL = Nodal marginal zone lymphoma, CR = complete remission

2Cda = 2-chlorodeoxyadenosine, CHL = chlorambucil, CHOP = cyclophosphamide, doxorubicin, vincristine, prednisone, COP = cyclophosphamide, vincristine, prednisone

R = anti-CD20 (rituximab), C = cyclophosphamide, R-CHOP = rituximab, cyclophosphamide, doxorubicin, vincristine, prednisone, RF = rituximab, fludarabine, ESHAP = etoposide, methylprednisolone, cytarabine, cisplatin, R-ICE = rituximab, ifosfamide, carboplatin, etoposide, R-FMD = rituximab, fludarabine, mitoxantrone, dexamethasone

ABVD = Adriamycin, bleomycin, vinblastine, dacarbazine, ASCT = autologous stem cell transplantation, BDR = Bortezomib, rituximab

T-LGL = T-large granular lymphocytic lymphoma

T-NHL = T-non-hodgkin lymphoma.

**Fig 1:**
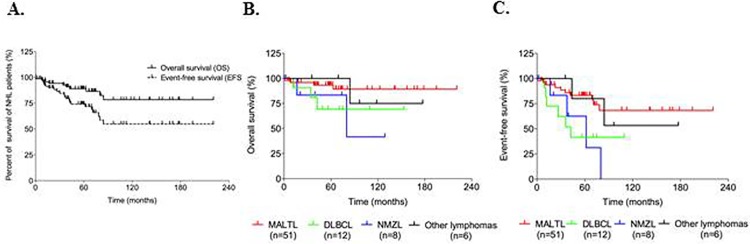
Survival of SS-associated lymphoma patients. **A.** Kaplan-Meier for OS and EFS for the entire cohort of SS-associated lymphoma patients. 5-year (%) OS for the entire lymphoma cohort: 90.91% (95%CI: 82.14–95.80%) and 5-year (%) EFS for the entire cohort: 77.92% (95% CI: 67.37–85.82%). **B.** Kaplan-Meier for OS of SS-associated lymphoma patients, according to lymphoma subtypes (log-rank p = 0.188). **C.** Kaplan-Meier for EFS of SS-associated lymphoma patients, according to lymphoma subtypes (log-rank test, P = 0.015).

At 5 years, OS and EFS for the entire NHL cohort was 90.91% (95%CI: 82.14–95.80%) and 77.92% (95% CI: 67.37–85.82%), respectively (Fig. 1A). According to NHL subtypes, the actuarial 5-year OS for SS patients with MALTL was 94.12% (95%CI: 83.46–98.59%), as opposed to 87.5% (95%CI: 50.78–99.89%) for those with NMZL, and 75.0% (95%CI: 46.15–91.73%) for patients with DLBCL; the rate for all other lymphomas was 100% (Fig. 1B). At 5 years, the actuarial EFS was 86.27% (95%CI: 76.50–96.05%) for patients with MALTL, 62.5% (95%CI: 30.38–86.51%) for those with NMZL, 50.0% (95%CI: 16.82–83.18%) for those with DLBCL, and 83.3% (95%CI: 41.78–98.86%) for those with other lymphomas (Fig. 1C). Survival curves of SS-associated lymphoma subgroups differed significantly for EFS (log rank p = 0.015) but not for OS (p = 0.188) (Fig. 1B and 1C). Patients with DLBCL had significantly poorer 5-year OS (p = 0.043) and 5-year EFS (p = 0.012) and a marginally increased risk (OR = 1.578, 95%CI: 0.983–2.533, p = 0.059) to present an event during follow-up, compared to MALTL patients (Table 4).

**Table 4:** Effects of prognostic parameters at the point of lymphoma diagnosis in SS-associated NHL patients’ outcomes.

	Deaths				Overall survival			
Parameters	OR (95%CI)	P-value	OR adjusted (95%CI)	P-value	HR (95%CI)	P-value	HR adjusted (95%CI)	P-value
NHL subtype	1.558 (0.865–2.804)	0.139	1.177 (0.568–2.440)	0.662	1.329 (0.806–2.193)	0.265	0.920 (0.511–1.655)	0.781
BM involvement (yes vs. no)	1.961 (0.493–7.791)	0.339	0.844 (0.168–4.234)	0.836	2.457 (0.689–8.759)	0.166	0.982 (0.243–3.967)	0.980
Total ESSDAI score (high vs. total)	5.241 (1.034–26.568)	0.045	2.367 (0.368–15.237)	0.364	5.946 (1.259–28.077)	0.024	2.451 (0.442–13.601)	0.305
IPI score (high/high-intermediate vs. low-intermediate/low)	13.867 (2.656–72.387)	0.002	9.628 (1.374–67.458)	0.023	11.414 (2.414–53.974)	0.002	8.529 (1.325–54.904)	0.024
	**Events**				**Event-free survival**			
**Parameters**	**OR (95%CI)**	**P-value**	**OR adjusted (95%CI)**	**P-value**	**HR (95%CI)**	**P-value**	**HR adjusted (95%CI)**	**P-value**
NHL subtype	1.578 (0.983–2.533)	0.059	1.230 (0.671–2.256)	0.503	1.288 (0.919–1.804)	0.142	0.845 (0.566–1.261)	0.409
BM involvement (yes vs. no)	3.333 (1.146–9.699)	0.027	1.700 (0.467–6.187)	0.421	3.789 (1.610–8.920)	0.002	1.646 (0.632–4.288)	0.308
Total ESSDAI score (high vs. total)	4.317 (1.460–12.771)	0.008	2.334 (0.641–8.500)	0.199	4.541 (1.772–11.637)	0.002	2.092 (0.722–6.064)	0.174
IPI score (high/high-intermediate vs. low-intermediate/low)	12.589 (3.911–40.526)	<0.001	6.996 (1.803–27.146)	0.005	8.718 (3.477–21.858)	<0.001	6.002 (1.791–20.114)	0.004

**Abbreviations**: NHL = Non-Hodgkin lymphoma, ESSDAI = EULAR Sjögren's syndrome disease activity index, IPI = International Prognostic Index

BM = Bone Marrow, OR = Odd ratio, HR = Hazard ratio, 95%CI = 95% confidence intervals.

### Predictors of NHL prognosis

**Fig 2:**
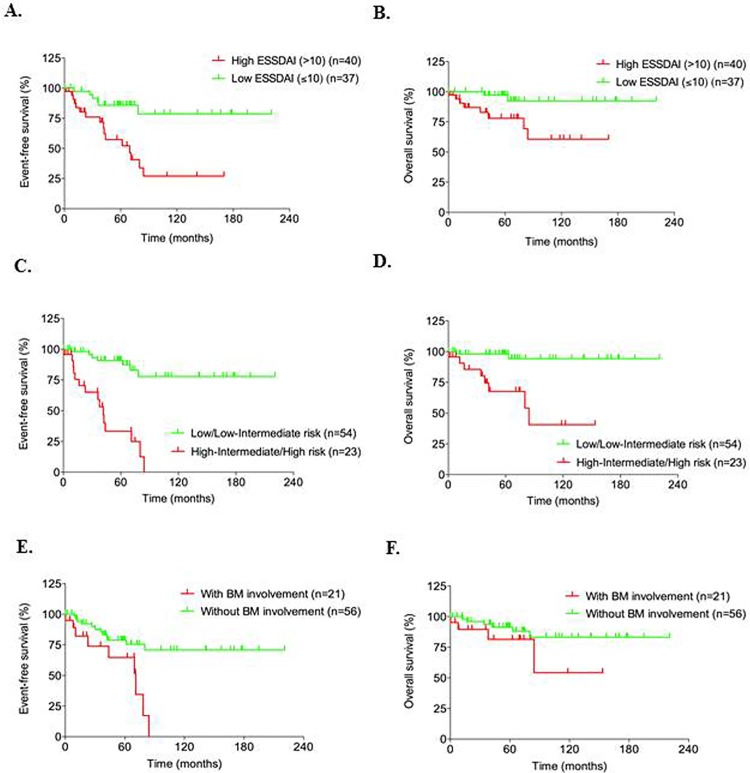
High SS disease activity, adverse IPI score and bone marrow involvement impair survival of lymphoma patients. A. and B. Kaplan-Meier for EFS and OS of SS-associated lymphoma patients, according to high or low total ESSDAI score groups (High total ESSDAI>10 and Low total ESSDAI≤10). High vs. Low total ESSDAI group for EFS: log-rank p = 0.001 and for OS: log-rank p = 0.011. C. and D. Kaplan-Meier for EFS and OS of the SS-associated NHL patients, according to IPI score groups: low/low-intermediate IPI group = 0–2 factors and high-intermediate/high IPI group = 3–4 factors. Log-rank test of low/low-intermediate vs. high-intermediate/high risk group curves for EFS: p< 0.001 and for OS: p<0.001. E. and F. Kaplan-Meier for EFS and OS of the SS-associated NHL patients, according to bone marrow involvement (log-rank test for EFS: p = 0.001 and for OS: p = 0.152).

Patients with high SS-disease activity (high total ESSDAI score >10) had greater risk to experience a death (OR = 5.241, 95%CI: 1.034–26.568, p = 0.045) or an event (OR = 4.317, 95%CI: 1.146–9.699, p = 0.008), and significantly worse EFS and OS compared to low total ESSDAI scored patients (score ≤10) (EFS: log-rank p = 0.001, HR = 4.541, 95%CI: 1.772–11.637; OS: log-rank p = 0.011, HR = 5.946, 95%CI: 1.259–28.077) (Fig. 2A and Fig. 2B) (Table 4). Moreover, the improvement in the total ESSDAI score six months after completion of first-line treatment (delta ESSDAI) among patients who had experienced an event (mean delta ESSDAI±SD: 4.59±1.68) was significantly less than that seen in event-free patients (mean delta ESSDAI±SD: 6.87±3.33) (p = 0.005).

Focusing further, we recognized additional predictors of lymphoma prognosis, such as an IPI score and BM involvement. In high/high-intermediate IPI group of patients, the risk of death was 13.867 times greater (95%CI: 2.656–72.387, p = 0.002) and the risk of event was 12.589 times greater (95%CI: 3.911–40.526, p<0.001) compared to low/low-intermediate IPI risk group (Table 4). An unfavorable IPI score (high-intermediate/high) was associated with worse EFS (log-rank p< 0.001, HR = 8.718, 95%CI: 3.477–21.858), as well as with worse OS (log-rank p<0.001, HR = 11.414, 95%CI: 2.414–53.974) compared to low-scored IPI groups (low/low-intermediate group) (Fig. 2C and 2D, Table 4). Actuarial 5-year OS and EFS for low/low-intermediate IPI risk group of SS-associated NHL patients was significant higher compared to high-intermediate/high IPI risk group (5-year OS%: 98.15%, 95% CI: 89.31–99.99% vs. 73.91%, 95% CI: 54.50–93.33%, p = 0.002 and 5-year EFS%: 92.59%, 95%CI: 85.38 99.81%, vs. 43.48%, 95%CI: 21.56–65.40%, p = 0.001). Combining Ann Arbor staging with the dissemination of MALTL, we observed that patients with limited disease (stage I-II) and those with disseminated disease (stage IV) but without BM involvement presented higher survival times compared with patients with advanced disease (stage IV) and BM involvement. Therefore, we targeted BM involvement as prognostic predictor of clinical outcome. SS-associated NHL patients with BM involvement at lymphoma diagnosis had 3.333 times greater risk (95%CI: 1.146–9.699, p = 0.027) to experience an event during follow-up (Table 4). Survival analyses revealed significantly poorer EFS (log-rank p = 0.001, HR: 3.789, 95%CI: 1.610–8.920) (Fig. 2E) without affecting mortality (log-rank p = 0.152) (Fig. 2F and Table 4).

After adjustment for identified independent prognostic parameters at the point of NHL diagnosis (lymphoma subtype, ESSDAI, IPI and BM involvement), only high IPI score retains a significantly increased risk for experiencing of event (adjusted OR = 6.996, 95%CI: 1.803–27.146, p = 0.005) or death (adjusted OR = 9.628, 95%CI: 1.374–67.458, p = 0.023). In our multivariate Cox regression model, high IPI score is associated with worse OS (adjusted HR = 8.529, 95%CI: 1.325–54.904, p = 0.024) and EFS (adjusted HR = 6.002, 95%CI: 1.791–20.114; p = 0.004) (Table 4). Among the other parameters, high total ESSDAI score retains the strongest trend for developing an event or a death during follow-up (Table 4) but no significance was reached, probably due to the small sample size.

## Discussion

To our knowledge, this is the largest single center study so far to systematically evaluate the impact of SS disease activity on the clinical course of SS-associated lymphomas [3,13,16–21,30,31]. The NHLs in this study were primarily MALTLs. Salivary glands were the most frequently involved extranodal site. Systemic organ involvement such as skin vasculitis, peripheral nerve involvement, anemia, lymphopenia, monoclonal immunoglobulin and cryoglobulinemia was more frequent among patients with an SS-associated lymphoma than in the general SS population, confirming previous observations [4,7,13,14,21].

The primary objective of this study was to identify prognostic risk factors for an adverse outcome and especially death in SS patients after lymphoma diagnosis. Several previous studies focused on identifying predictive markers for lymphoma development in SS patients. Specifically, those presenting with parotidomegaly, vasculitic purpura, splenomegaly, lymphadenopathy, cryoglobulinemia, low C4 levels, neutropenia and lymphopenia at SS diagnosis were seen to have a much greater probability of developing lymphoma and are hence considered as high-risk patients [1–4,6,8,13,14,19,32]. The present study has taken this one step further in demonstrating that all these markers further denote SS-associated lymphoma outcome. The ESSDAI score is a useful and objective instrument for SS activity quantification. It has also been shown to correlate with B cell biomarkers of disease activity such as beta-2 microglobulin, serum free light chains of immunoglobulins, the cytokine BAFF and FMS-like tyrosine kinase 3 ligand levels, as well as with cytopenias, hypocomplementemia and cryoglobulinemia [33–36]. High-score ESSDAI lymphoma patients in our study had significantly worse survival compared with those scoring a low ESSDAI. Additionally, the change/improvement in the total ESSDAI six months after completion of chemotherapy (delta ESSDAI) in patients who experienced an event was significantly lower than that seen in event-free patients. Consequently, although the ESSDAI score has not yet proved to be able to predict lymphoma development, it is an excellent prognostic marker for SS-associated lymphoma outcome. The simplicity of its application combined with its prognostic value lends ESSDAI a key role in lymphoma monitoring and management. These results are in line with the data published by Pollard et al. who suggested the possible implication of SS disease activity in the outcome of SS-associated MALTLs [30].

Our prognostic analysis also incorporated clinical features that reflect the growth and invasive potential of the tumor. Since its implementation in1993 in patients with aggressive lymphoma, IPI has been extensively applied to all types of NHL, including indolent lymphomas [26,37]. According to our study, SS-associated lymphoma patients with a high IPI-score (IPI = 3–4 factors) had a worse prognosis compared to those with a low score (IPI = 0–2 factors). Our cohort demonstrated similar 5-year OS, 5-year EFS and CR rates for non-gastric MALTLs as those reported for the general population [38–42]. Among SS-associated MALTL patients with a median follow-up of 58 months, the 5-year OS, 5-year EFS and CR were comparable with those described by Zucca et al. for non-gastric MALTLs in the general population [40]. Despite the fact that 20% of SS patients with MALTL in our study presented with Stage IV disease with multiple MALT organ localizations, their outcome remains unaffected by the multi-focal nature of the lymphoma (5-year survival of 90%). Interestingly, these observations were first made by Thieblemont et al. in 158 patients with MALTLs and were subsequently confirmed in another larger cohort of 180 non-gastric MALTL patients in the general population. In the latter study, BM involvement was associated with a worse prognosis [39,40]. Equally, the presence of BM involvement in our study was prognostic of an impaired clinical outcome. Nevertheless, the similarity in the behavior between SS-MALTLs and MALTLs in the general population is somewhat surprising. One might hypothesize that the continuous persistence of the inflammatory drive initially leading to the development of lymphoma would also accelerate relapses as compared to patients in whom this drive was eliminated. However, our findings at a median follow-up of 58 months could not support this hypothesis. This may possibly be explained by the fact that previous studies with MALTLs in the general population included a substantial number of patients with autoimmune diseases, which might have affected the EFS of the entire lymphoma cohort [39,40,43,44].

Mainly because of its retrospective setting, our study had several limitations. ESSDAI was established relatively lately and published in 2010. In this respect, for the majority of patients, ESSDAI was estimated retrospectively at the time of SS and at the time of lymphoma diagnosis as well as 6 months after lymphoma treatment. Despite the high rate of data completeness of our study, the quality of ESSDAI has not been demonstrated yet when it is used retrospectively. Before lymphoma development, ESSDAI score was retrospectively evaluated in only one distant time-point, at SS diagnosis, affecting the dynamic character of disease activity scoring scale. In addition, some features included in the ESSDAI, such as hypocomplementemia, cryoglobulins, glandular swelling are known risk factors for lymphoma development and doubtlessly introduced further bias in the demonstration of a worse survival in those patients with original high ESSDAI score. On the other hand, at SS diagnosis, total ESSDAI score showed no significant differences between SS cases that latterly developed or not lymphoma. This finding is in agreement with a recent study by Risselada et al, where ESSDAI score could not clearly discriminate between patients with or without subsequent lymphoma development [45]. Ideally, measurement of ESSDAI in the year or 2 years before NHL diagnosis would be better to assess the potential predictive role of SS activity to lymphoma development.

## Conclusions

We detected that ESSDAI and IPI scores are valuable prognostic parameters of SS-associated NHL outcome. Validation of these prognostic factors through a prospective cohort will enable clinicians to treat SS-lymphoma patients with a more patient-tailored approach.
